# Supplementary material for: Assessing roost disturbance of straw-coloured fruit bats (Eidolon helvum) through tri-axial acceleration
Source: PLoS One. 2020 Nov 23;15(11):e0242662. doi: 10.1371/journal.pone.0242662 (PMC7682868; doi:10.1371/journal.pone.0242662)
Supplement: S1 File — (PDF) [file pone.0242662.s001.pdf]

Supporting Information

Table S1. Metadata of the tracking dataset. Animal ID: animal identity code; Day 1: first day of tracking; Days: number of days of tracking; Sex: male (m) or female (f); Age: adult (ad) or young adult (yg-ad); Forearm: forearm length (mm); BM: body mass (g); Attachment: datalogger attachment method; TM: tag mass (sum of datalogger and collar mass in g); ACC burst: acceleration burst duration per minute (s); ACC freq: accelerometer frequency (Hz).

Animal ID	Location	Year	Season	Day 1	Days	Sex	Age	Forearm	BM	Attachment	TM	ACC burst	ACC freq
1079	Accra	2009	wet	27-Aug	2	m	ad	118.1	284	glue	19.5	14	18.74
1080	Accra	2009	wet	27-Aug	1	m	ad	113.9	244	glue	19.5	14	18.74
1081	Accra	2009	wet	27-Aug	2	m	ad	123.5	274	glue	19.5	14	18.74
1084	Accra	2009	wet	29-Aug	3	m	ad	115.1	239	glue	19.5	14	18.74
1086	Accra	2009	wet	29-Aug	1	m	ad	118.2	277	glue	19.5	14	18.74
1088	Accra	2009	wet	29-Aug	1	m	ad	120	247	glue	19.5	14	18.74
1607	Accra	2011	dry	04-Feb	5	m	ad	124.7	321	glue	25.5	14	18.74
1616	Accra	2011	dry	06-Feb	1	m	ad	121	292	glue	25.5	14	18.74
1620	Accra	2011	dry	07-Feb	1	m	ad	119.7	255	glue	20	14	18.74
1626	Accra	2011	dry	03-Feb	1	m	ad	119.1	280	glue	20.5	14	18.74
1875	Kibi	2011	wet	28-Aug	2	f	ad	119.1	280	glue	21.1	14	18.74

1870_2	Kibi	2011	wet	30-Aug	1	m	ad	117.5	275	glue	21.5	14	18.74
2394	Kibi	2012	wet	10-Sep	1	m	ad	118.8	275	collar	25.4	14	18.74
2396	Kibi	2012	wet	29-Aug	5	m	ad	121.6	270	collar	24.3	14	18.74
2402	Kibi	2012	wet	07-Sep	5	m	ad	120.3	272	collar	23	14	18.74
2404	Kibi	2012	wet	14-Sep	3	m	yg-ad	121.9	270	collar	23.5	14	18.74
2608	Kibi	2013	dry	26-Jan	3	m	ad	117.9	275	collar	26.2	14	18.74
2612	Kibi	2013	dry	26-Jan	3	m	ad	125.6	250	collar	25.9	14	18.74
2772	Kibi	2013	dry	31-Jan	2	m	ad	122	245	collar	25.8	14	18.74
2609	Kibi	2013	wet	21-Sep	4	m	ad	122.5	290	collar	22.2	14	18.74
1618	Ouagadougou	2013	wet	20-Aug	5	m	ad	103.7	250	collar	25	14	18.74
1619	Ouagadougou	2013	wet	20-Aug	6	m	ad	118.9	275	collar	25	14	18.74
1621	Ouagadougou	2013	wet	29-Aug	3	m	ad	124	260	collar	25	14	18.74
1624	Ouagadougou	2013	wet	22-Aug	4	m	ad	124.8	280	collar	25	14	18.74
3967	Ouagadougou	2014	wet	18-Jun	2	m	ad	121.4	280	collar	25	14	18.74
3969	Ouagadougou	2014	wet	19-Jun	4	m	ad	116.6	270	collar	25	14	18.74
3970	Ouagadougou	2014	wet	19-Jun	4	m	ad	122.4	300	collar	25	14	18.74
3971	Ouagadougou	2014	wet	19-Jun	5	m	ad	121.3	315	collar	25	14	18.74
3972	Ouagadougou	2014	wet	20-Jun	1	m	ad	122	255	collar	25	14	18.74

3973	Ouagadougou	2014	wet	20-Jun	4	m	ad	123.7	265	collar	25	14	18.74
3359	Kasanka	2013	wet	05-Dec	5	m	yg-ad	126.1	290	collar	23	14	18.74
3364	Kasanka	2013	wet	06-Dec	5	m	ad	118.1	285	collar	24	14	18.74
3370	Kasanka	2013	wet	06-Dec	4	m	ad	124	298	collar	23	14	18.74
4148	Kasanka	2014	wet	30-Nov	4	m	ad	131.2	306	collar	23.5	13	20
4149	Kasanka	2014	wet	30-Nov	6	m	ad	116.4	278	collar	23.5	13	20
4151	Kasanka	2014	wet	30-Nov	7	m	ad	125.4	275	collar	23.5	13	20
4154	Kasanka	2014	wet	30-Nov	6	m	ad	122.3	269	collar	23.5	13	20
4155	Kasanka	2014	wet	30-Nov	6	m	ad	120.6	282	collar	23.5	13	20
4156	Kasanka	2014	wet	01-Dec	6	m	ad	125.1	278	collar	23.5	13	20
4157	Kasanka	2014	wet	01-Dec	6	m	ad	126.4	315	collar	23.5	13	20
4158	Kasanka	2014	wet	01-Dec	6	m	ad	121.4	274	collar	23.5	13	20
4160	Kasanka	2014	wet	30-Nov	6	m	ad	127.3	286	collar	23.5	13	20
4161	Kasanka	2014	wet	30-Nov	1	m	ad	122.9	272	collar	23.5	13	20
4162	Kasanka	2014	wet	01-Dec	1	m	ad	124.6	293	collar	23.5	13	20
4163	Kasanka	2014	wet	01-Dec	7	m	ad	121.7	273	collar	23.5	13	20
4164	Kasanka	2014	wet	01-Dec	6	m	ad	121.2	281	collar	23.5	13	20

Table S2. Roost location for each animal in each tracking day. Animal ID: animal identity code; Date: date of the tracking day; Latitude and longitude: coordinates of the roost used by the bat in that tracking day.

Animal ID	Location	Date	Latitude	Longitude
1079	Accra	27-08-2009	5.585982	-0.183093
1079	Accra	28-08-2009	5.585982	-0.183093
1080	Accra	27-08-2009	5.585982	-0.183093
1081	Accra	27-08-2009	5.585982	-0.183093
1081	Accra	28-08-2009	5.585982	-0.183093
1084	Accra	29-08-2009	5.585982	-0.183093
1084	Accra	30-08-2009	5.585982	-0.183093
1084	Accra	31-08-2009	5.585982	-0.183093
1086	Accra	29-08-2009	5.585982	-0.183093
1088	Accra	29-08-2009	5.585982	-0.183093
1607	Accra	04-02-2011	5.585982	-0.183093
1607	Accra	05-02-2011	5.585982	-0.183093
1607	Accra	06-02-2011	5.585982	-0.183093
1607	Accra	07-02-2011	5.585982	-0.183093
1607	Accra	08-02-2011	5.585982	-0.183093

1616	Accra	06-02-2011	5.585982	-0.183093
1620	Accra	07-02-2011	5.585982	-0.183093
1626	Accra	03-02-2011	5.585982	-0.183093
1875	Kibi	28-08-2011	6.16505	-0.55533
1875	Kibi	29-08-2011	6.16505	-0.55533
1870_2	Kibi	30-08-2011	5.585982	-0.183093
2394	Kibi	10-09-2012	6.39352	-0.54314
2396	Kibi	29-08-2012	6.23441	-0.39391
2396	Kibi	30-08-2012	6.23441	-0.39391
2396	Kibi	31-08-2012	6.23441	-0.39391
2396	Kibi	01-09-2012	6.23441	-0.39391
2396	Kibi	02-09-2012	6.23441	-0.39391
2402	Kibi	07-09-2012	6.253297	-0.439378
2402	Kibi	08-09-2012	6.23441	-0.39391
2402	Kibi	09-09-2012	6.23441	-0.39391
2402	Kibi	10-09-2012	6.23441	-0.39391
2402	Kibi	11-09-2012	6.23441	-0.39391
2404	Kibi	14-09-2012	6.551665	-0.321632

2404	Kibi	15-09-2012	6.39352	-0.54314
2404	Kibi	16-09-2012	6.39352	-0.54314
2608	Kibi	26-01-2013	6.22709	-0.35778
2608	Kibi	27-01-2013	6.23441	-0.39391
2608	Kibi	28-01-2013	6.23441	-0.39391
2612	Kibi	26-01-2013	6.221674	-0.424875
2612	Kibi	27-01-2013	6.221674	-0.424875
2612	Kibi	28-01-2013	5.585982	-0.183093
2772	Kibi	31-01-2013	6.23441	-0.39391
2772	Kibi	01-02-2013	6.23441	-0.39391
2609	Kibi	21-09-2013	6.23441	-0.39391
2609	Kibi	22-09-2013	6.23441	-0.39391
2609	Kibi	23-09-2013	6.23441	-0.39391
2609	Kibi	24-09-2013	6.23441	-0.39391
1618	Ouagadougou	20-08-2013	12.397233	-1.491938
1618	Ouagadougou	21-08-2013	12.58846	-1.38655
1618	Ouagadougou	22-08-2013	12.58846	-1.38655
1618	Ouagadougou	23-08-2013	12.58846	-1.38655

1618	Ouagadougou	24-08-2013	12.58846	-1.38655
1619	Ouagadougou	20-08-2013	12.47313	-1.43262
1619	Ouagadougou	21-08-2013	12.397233	-1.491938
1619	Ouagadougou	22-08-2013	12.397233	-1.491938
1619	Ouagadougou	23-08-2013	12.397233	-1.491938
1619	Ouagadougou	24-08-2013	12.397233	-1.491938
1619	Ouagadougou	25-08-2013	12.397233	-1.491938
1621	Ouagadougou	29-08-2013	12.447213	-1.271226
1621	Ouagadougou	30-08-2013	12.397233	-1.491938
1621	Ouagadougou	31-08-2013	12.397233	-1.491938
1624	Ouagadougou	22-08-2013	12.40645	-1.68505
1624	Ouagadougou	23-08-2013	12.33754	-1.82931
1624	Ouagadougou	24-08-2013	12.20097	-1.90395
1624	Ouagadougou	25-08-2013	12.20097	-1.90395
3967	Ouagadougou	18-06-2014	12.397233	-1.491938
3967	Ouagadougou	19-06-2014	12.397233	-1.491938
3969	Ouagadougou	19-06-2014	12.397233	-1.491938
3969	Ouagadougou	20-06-2014	12.397233	-1.491938

3969	Ouagadougou	21-06-2014	12.397233	-1.491938
3969	Ouagadougou	22-06-2014	12.51418	-1.55509
3970	Ouagadougou	19-06-2014	12.397233	-1.491938
3970	Ouagadougou	20-06-2014	12.397233	-1.491938
3970	Ouagadougou	21-06-2014	12.397233	-1.491938
3970	Ouagadougou	22-06-2014	12.397233	-1.491938
3971	Ouagadougou	19-06-2014	12.397233	-1.491938
3971	Ouagadougou	20-06-2014	12.397233	-1.491938
3971	Ouagadougou	21-06-2014	12.397233	-1.491938
3971	Ouagadougou	22-06-2014	12.397233	-1.491938
3971	Ouagadougou	23-06-2014	12.397233	-1.491938
3972	Ouagadougou	20-06-2014	12.397233	-1.491938
3973	Ouagadougou	20-06-2014	12.397233	-1.491938
3973	Ouagadougou	21-06-2014	12.48092	-1.3911
3973	Ouagadougou	22-06-2014	12.48092	-1.3911
3973	Ouagadougou	23-06-2014	12.58012	-1.23463
3359	Kasanka	05-12-2013	-12.404971	29.78388
3359	Kasanka	06-12-2013	-12.58687	30.24232

3359	Kasanka	07-12-2013	-12.58687	30.24232
3359	Kasanka	08-12-2013	-12.58687	30.24232
3359	Kasanka	09-12-2013	-12.58687	30.24232
3364	Kasanka	06-12-2013	-12.36104	29.97449
3364	Kasanka	07-12-2013	-12.48308	30.12932
3364	Kasanka	08-12-2013	-12.50457	30.19164
3364	Kasanka	09-12-2013	-12.58687	30.24232
3364	Kasanka	10-12-2013	-12.85819	30.51915
3370	Kasanka	06-12-2013	-12.58687	30.24232
3370	Kasanka	07-12-2013	-12.58687	30.24232
3370	Kasanka	08-12-2013	-12.58687	30.24232
3370	Kasanka	09-12-2013	-12.58687	30.24232
4148	Kasanka	30-11-2014	-12.58687	30.24232
4148	Kasanka	01-12-2014	-12.58687	30.24232
4148	Kasanka	02-12-2014	-12.58687	30.24232
4148	Kasanka	03-12-2014	-12.58687	30.24232
4149	Kasanka	30-11-2014	-12.58687	30.24232
4149	Kasanka	01-12-2014	-12.58687	30.24232

4149	Kasanka	02-12-2014	-12.58687	30.24232
4149	Kasanka	03-12-2014	-12.58687	30.24232
4149	Kasanka	04-12-2014	-12.58687	30.24232
4149	Kasanka	05-12-2014	-12.58687	30.24232
4151	Kasanka	30-11-2014	-12.58687	30.24232
4151	Kasanka	01-12-2014	-12.58687	30.24232
4151	Kasanka	02-12-2014	-12.58687	30.24232
4151	Kasanka	03-12-2014	-12.58687	30.24232
4151	Kasanka	04-12-2014	-12.58687	30.24232
4151	Kasanka	05-12-2014	-12.58687	30.24232
4151	Kasanka	06-12-2014	-12.58687	30.24232
4154	Kasanka	30-11-2014	-12.7838	30.52498
4154	Kasanka	01-12-2014	-12.58687	30.24232
4154	Kasanka	02-12-2014	-12.58687	30.24232
4154	Kasanka	03-12-2014	-12.58687	30.24232
4154	Kasanka	04-12-2014	-12.7838	30.52498
4154	Kasanka	05-12-2014	-12.58687	30.24232
4155	Kasanka	30-11-2014	-12.727	30.31176

4155	Kasanka	01-12-2014	-12.58687	30.24232
4155	Kasanka	02-12-2014	-12.58687	30.24232
4155	Kasanka	03-12-2014	-12.58687	30.24232
4155	Kasanka	04-12-2014	-12.58687	30.24232
4155	Kasanka	05-12-2014	-12.58687	30.24232
4156	Kasanka	01-12-2014	-12.72403	30.57081
4156	Kasanka	02-12-2014	-12.58687	30.24232
4156	Kasanka	03-12-2014	-12.58687	30.24232
4156	Kasanka	04-12-2014	-12.58687	30.24232
4156	Kasanka	05-12-2014	-12.58687	30.24232
4156	Kasanka	06-12-2014	-12.65657	30.52616
4157	Kasanka	01-12-2014	-12.58687	30.24232
4157	Kasanka	02-12-2014	-12.58687	30.24232
4157	Kasanka	03-12-2014	-12.58687	30.24232
4157	Kasanka	04-12-2014	-12.58687	30.24232
4157	Kasanka	05-12-2014	-12.58687	30.24232
4157	Kasanka	06-12-2014	-12.58687	30.24232
4158	Kasanka	01-12-2014	-12.58687	30.24232

4158	Kasanka	02-12-2014	-12.58687	30.24232
4158	Kasanka	03-12-2014	-12.58687	30.24232
4158	Kasanka	04-12-2014	-12.58687	30.24232
4158	Kasanka	05-12-2014	-12.58687	30.24232
4158	Kasanka	06-12-2014	-12.58687	30.24232
4160	Kasanka	30-11-2014	-12.58687	30.24232
4160	Kasanka	01-12-2014	-12.58687	30.24232
4160	Kasanka	02-12-2014	-12.58687	30.24232
4160	Kasanka	03-12-2014	-12.58687	30.24232
4160	Kasanka	04-12-2014	-12.58687	30.24232
4160	Kasanka	05-12-2014	-12.58687	30.24232
4161	Kasanka	30-11-2014	-12.43961	30.4753
4162	Kasanka	01-12-2014	-12.58687	30.24232
4163	Kasanka	01-12-2014	-12.58687	30.24232
4163	Kasanka	02-12-2014	-12.73757	30.54503
4163	Kasanka	03-12-2014	-12.73757	30.54503
4163	Kasanka	04-12-2014	-12.73757	30.54503
4163	Kasanka	05-12-2014	-12.73757	30.54503

4163	Kasanka	06-12-2014	-12.58687	30.24232
4163	Kasanka	07-12-2014	-12.73757	30.54503
4164	Kasanka	01-12-2014	-12.58687	30.24232
4164	Kasanka	02-12-2014	-12.58687	30.24232
4164	Kasanka	03-12-2014	-12.58687	30.24232
4164	Kasanka	04-12-2014	-12.58687	30.24232
4164	Kasanka	05-12-2014	-12.58687	30.24232
4164	Kasanka	06-12-2014	-12.58687	30.24232

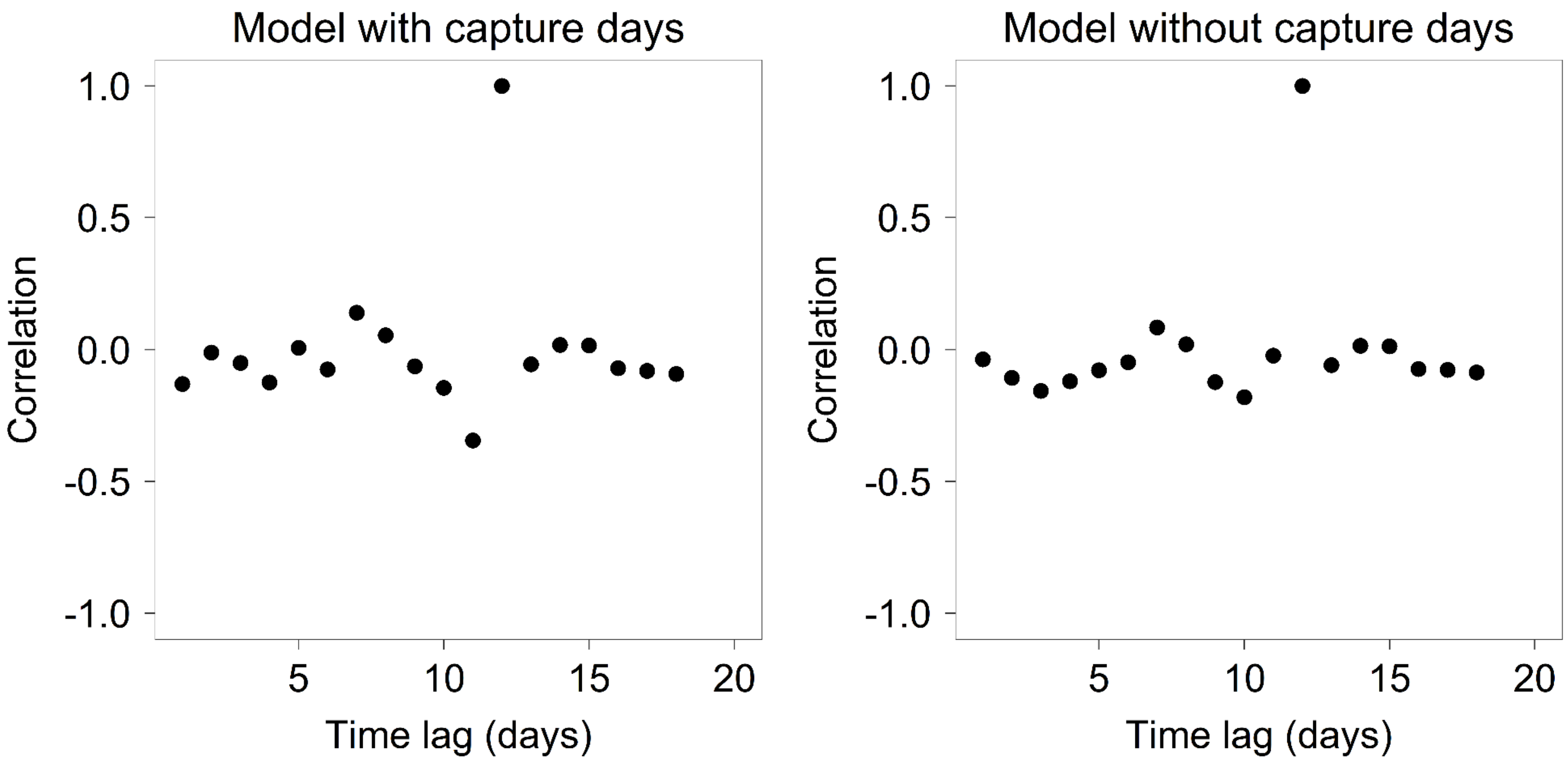

Figure S1. Temporal autocorrelation of the GLMMs residuals. Left panel: residual autocorrelation for the model using the full data set; Right panel: residual autocorrelation for the model that excluded trapping days. The function acf of the stats R-package [1] was used to compute temporal correlations.

References

1. R Core Team. R: A language and environment for statistical computing. Vienna, Austria: R Foundation for Statistical Computing; 2018. http://www.R-project.org/
