# Supplementary material for: Assessing roost disturbance of straw-coloured fruit bats (Eidolon helvum) through tri-axial acceleration
Source: PLoS One. 2020 Nov 23;15(11):e0242662. doi: 10.1371/journal.pone.0242662 (PMC7682868; doi:10.1371/journal.pone.0242662)
Supplement: Attachment — Submitted filename: Response to Reviewers.pdf [file pone.0242662.s003.pdf]

Reviewer #3 comments

Thank you for the revised version of your manuscript (previously Reviewer 3). As before, this study assesses roost disturbance in straw-coloured fruit bats using GPS-accelerometers to measure the probability of daytime flights using the bat capture days as a proxy for human disturbance. The statistics are much improved without the use of the human density metric which was the same at each colony. There are still a few question marks I have over the analysis, particularly in relation to roost ID (see below), but I do not think these will have much impact on the results. I congratulate the authors on a well-written manuscript.

Minor comments:

The authors have used roost ID rather than colony as a random effect, which means for some colonies there are multiple roosts and others just the main colony site. For example, Ouagadougou seems to have 10 roosts whereas has Accra only has the main colony site. Although designed to reduce the bias of repeated measures, this design could give additional weight to certain colonies, particularly when you consider some of the weather data for each roost is taken from the same weather station (e.g. bats at different roosts on the same day would have the same solar irradiance value). I think any impacts would be very minor, and I also think you need to take the bat biology into account (e.g. significance of different roosts), which I am not able to do as I'm not a bat expert. It would be helpful if you could run your analyses using colony location as well and/or justify your decision to use roost in the manuscript when you refer to colony in the rest of the manuscript.

Response: We think we should account for possible roost specific effects on bat behaviour, which would not be accounted if we use colony site as random effect instead. Please note in Fig. 1 that roosts can be as far as 90 km from each other, thus potentially under different environmental conditions. Colony sites, on the other hand, do not have as much ecological meaning as they simply mark the places where the captures were conducted.
We anyway ran the analysis with colony site as random effect, as suggested, and the results were nearly identical (see the table below; the legend is the same as for Table 2 in the manuscript).

Model	Parameter	Range	Estimate	SE	*Z*	P-value	R^2 cond./marg
With trapping days	Intercept	-	-7.169	2.033	-3.53	>0.001	0.24/0.22
	Trapping day	0-1	1.666	0.530	3.14	**0.002**	
	Solar irradiance	2.5-30.7	0.242	0.090	2.68	**0.007**	
	Precipitation	0.0-87.0	0.042	0.043	0.99	0.329	
	Wind speed	0.5-4.0	-0.320	0.377	-0.85	0.398	
Without trapping days	Intercept	-	-6.008	1.831	-3.28	0.001	0.06/0.06
	Solar irradiance	2.5-30.7	0.175	0.089	1.96	**0.050**	
	Precipitation	0.0-87.0	0.034	0.042	0.82	0.410	
	Wind speed	0.5-4.0	-0.144	0.429	-0.34	0.738	

We agree that we used the term “colony” several times when we should have used “roost”. That was corrected across the manuscript.

L55: has = have

Response: We think “has” should be used here as it refers to “The use of automated methods”.

Table 1: Please distinguish the two Kibi colonies as you refer to 5 colonies throughout the rest of the manuscript. Given that the two Kibi colonies are so close together and that the bats do seem to move roosts further than the distance between these two colonies, I’m not totally sure whether you should be referring to 4 or 5 colonies (when considering the spatial scale between these two sites and the other 3), but I’m not a bat expert so I leave this to you.

Response: We made the changes suggested in Table 1. We now refer to roosts instead of colonies (see above), thus we maintained the reference to 5, instead of 4.

L208: If you use Roost ID as a random effect, please add an additional supplementary table detailing the sample size at each roost (bats and days). I presume this cannot be added to Table S1

as some of the bats will have swapped roost, but it’s helpful to see the overall distribution of your sample size.

Response: We added a supplementary table (Table S2) containing the roost location for each bat in each day of tracking.
